# Preparation of an LZ-OEO Compound Antibacterial Gel and the Effect of Microwave Treatment on Its Structure and Stability

**DOI:** 10.3390/gels8120838

**Published:** 2022-12-19

**Authors:** Shuaishuai Wei, Ying Gao, Lulu Ma, Zhan Wang, Xin Liu, Ying Liu, Mengzhen Zhong, Shijian Dong, Shugang Li

**Affiliations:** 1 Engineering Research Center of Bio-Process, Ministry of Education, Key Laboratory for Agricultural Products Processing of Anhui Province/School of Food and Biological Engineering, Hefei University of Technology, Hefei 230601, China; 2 Key Laboratory of Fermentation Engineering, Ministry of Education, School of Food and Biological Engineering, Hubei University of Technology, Wuhan 430068, China; 3 Anhui Rongda Food Co., Ltd., Xuancheng 242000, China

**Keywords:** microwave treatment, lysozyme, oregano essential oil, antibacterial gel, gel stability

## Abstract

Composite gels prepared with ovalbumin (OVA) as basic materials have been gradually utilized in food and biological fields. However, the structure and function of gels made from natural materials are not perfect, especially the hardness, viscoelasticity and water-holding capacity of gels, which are easily affected by various factors (pH, NaCl, etc.). In order to improve the antibacterial effect and safety of gels, and on the basis of exploring the bacteriostatic formula of lysozyme-oregano essential oil (LZ-OEO), the influence of microwave treatment on the stability of the composite bacteriostatic material gel was emphatically investigated and discussed so as to develop a new bacteriostatic gel material. The results revealed that the LZ-OEO antibacterial gel prepared by adding 20% OEO, with a ratio of 3:2 between OVA and LZ, was more stable after microwave treatment, and the synergistic antibacterial effect was significantly improved. That is, the OVA and LZ-OEO composite gel processed using a 350 W microwave treatment for 1 min had the highest hardness, the water-holding capacity reached 78.05% and a dense and ordered network structure was formed. In addition, the compound gel displayed excellent antibacterial effects against *Staphylococcus aureus* and *Escherichia coli*. The experimental findings in this study effectively expands the application scope of lysozyme antibacterial materials and provides a more favorable technical foundation for future development and utilization.

## 1. Introduction

Gels, a type of polymer dispersion material mainly made up of either colloidal particles or different polymers cross-linked with each other, are considered an important functional property of edible food protein because gels possess great water-holding capacity and viscoelasticity [1,2]. The thermogenic gels made from ovalbumin (OVA) and inulin have shown advantages in texture, sensory properties, processing performance and nutritional value and are gradually adopted for use in the field of food biology [3,4,5,6]. However, due to its rich nutrition, the storage of gels easily suffers from bacterial infestation, spoilage and unstable quality [7]. Therefore, improving the antibacterial property of OVA composite gel is the key to expanding its application in food and biomedical fields.

In recent years, with the public’s enhanced awareness of health and safety, natural antibacterial agents have gradually become the more preferable choices because they inhibit the growth of bacteria and maintain health and safety, and they possess the properties of being safe, economical and environmentally friendly, drawing more and more of the public’s attention [8]. Lysozyme (LZ) is a natural peptide protein bacteriostatic agent, which was found to break the β-1,4 glycosidic bond between N-acetylcytidylic acid and N-acetylamino glucose, thereby decomposing the cell wall and inhibiting bacterial activity; for this reason, LZ can be added to packing materials to act as an inhibiting bacterial agent [9,10]. The main effects of LZ are limited to Gram-positive bacteria, while its effects on Gram-negative bacteria are not obvious. Therefore, it is necessary to combine LZ with other antibacterial agents to improve the overall antibacterial property of the composite gel. Oregano essential oil (OEO) is a natural aromatic amber-colored oily liquid extracted from the stems and leaves of the whole oregano plant. It contains aromatic phenols, terpenes, tannins, resins and other chemical components, which have strong bactericidal, bacteriostatic and antioxidant effects; [11,12,13,14] found that OEO significantly inhibited the growth of *Escherichia coli* (*E. coli*), a Gram-negative bacteria. Compared with other antibacterial materials, LZ and OEO have the advantages of being natural, safe and edible, and could be widely used in the food industry. Therefore, it is speculated that they might complement each other and play a synergistic antibacterial effect; consequently, LZ and OEO were selected as antibacterial materials.

However, OEO is a volatile essential oil and that might affect the stability of a composite gel to some extent [15]. Therefore, it was necessary to find a rational modification method to improve the stability. Microwave treatment is an effective physical technique that can affect the structure and function of proteins [16,17,18]. Zhu et al. found that microwave treatment could thaw meat; improve the water-holding capacity, color and dynamic rheological properties of meat; and contribute to the production of meat products [19]. The improvements of microwave treatment on water-holding capacity, rheological properties and gel strength were also found in soybean isolate-wheat gluten gels [20]. Hence, the microwave treatment on the effects of antibacterial gel is worth exploring.

Cheng et al. reported a series of all-small-molecule dynamic covalent gels with antibacterial properties, which was caused by direct gelation between tannic and inorganic borates or organic borates [21]. Recently, scientists have focused their efforts toward the development of self-assembled peptide and polymeric gels/hydrogels, as antibacterial biomaterials, to address the challenge of antibiotic resistance in bacteria [22]. The antibacterial agents could be entrapped within the gel matrix employing covalent bonds or noncovalent interactions. The biomaterials also exhibited great potential in antibacterial applications, such as for the prevention of biomaterial-related infections [23].

Therefore, the LZ-OEO antibacterial gel, compared to OVA composite gel, was produced in this project by the addition of LZ and OEO via microwave treatment. The gel effectively inhibited the growth of both Gram-positive and Gram-negative bacteria. The gel’s properties of water-holding capacity (WHC), apparent viscosity, rheology, hardness and distribution were improved by microwave treatment. The aim of this study was to develop a new antibacterial gel with good physical and chemical properties. Thus, the application of LZ-OEO bacteriostatic products would be improved. A new volatile essential oil stabilization technology system was constructed, which provides technical support for the development and utilization of LZ’s antibacterial effects and also provides a technical reference for the development and utilization of natural antibacterial essential oil with a strong antibacterial effect and volatility.

## 2. Results and Discussion

### 2.1. Study on the Antibacterial Gel Formulation of Lysozyme (LZ) and Oregano Essential Oil (OEO)

#### 2.1.1. Addition of LZ

**Figure 1:**
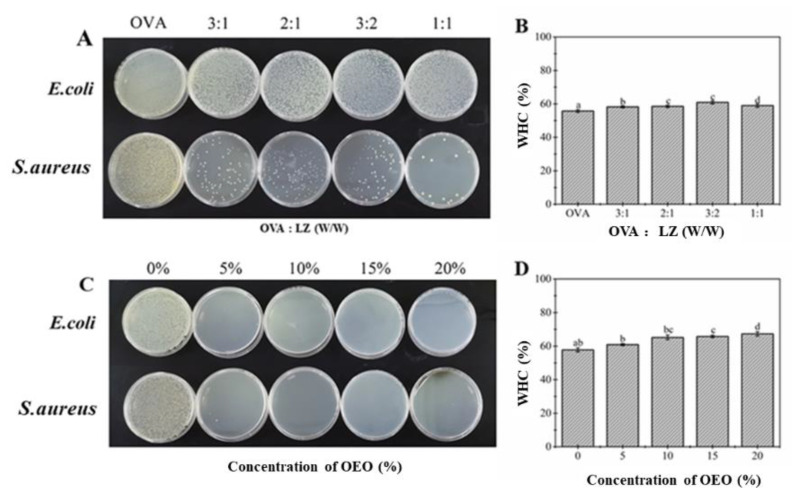
Effects of the addition ratio of lysozyme (LZ) (**A**,**B**) and oregano essential oil (OEO) (**C**,**D**) on the antibacterial properties and water-holding capacity of the composite gel. Different letters of a–d refer to statistically significant differences among the values (*p* < 0.05).

Bacteriostasis and water-holding capacity were treated as the main observation indicators. In order to determine the amount of LZ added to the OVA-IN composite gel, four composite gels with different addition ratios between OVA and LZ (3:1, 2:1, 3:2 and 1:1) were selected in this experiment, and the composite gel sample without the addition of LZ was taken as the control group. This explored the effect of the addition of LZ on the bacteriostasis and water-holding capacity of the above gel, as shown in Figure 1A. With an increasing amount of LZ, the growth of *Staphylococcus aureus* in the composite gel was significantly inhibited, indicating that the addition of LZ could make the composite gel exhibit a certain antibacterial effect. When the ratio of OVA to LZ was greater than 3:2, the inhibition effect on *Staphylococcus aureus* was no longer significantly displayed. However, the addition of LZ did not inhibit the growth of *Escherichia coli* in the composite gel. The key reason might lie in that the peptidoglycan content in Gram-negative bacteria (*Escherichia coli*) was comparatively low, and it was mainly embedded in the lipopolysaccharide layer of the outer membrane of the cell wall, which hindered the lysis of LZ. Therefore, LZ did not show an obvious inhibitory effect on *Escherichia coli* [24].

The impact of the addition of LZ on WHC of composite gel is shown in Figure 1B. The WHC was found to increase with a heavier dosage of LZ until the mass ratio of OVA to LZ reached 3:2 and then started to decrease [24]. The slight decrease of WHC might be related to how the excessive amount of LZ increased the protein concentration of the gel, prompted the exposure of hydrophobic groups and strengthened the interaction between protein molecules, and therefore formed a tight network structure with decreased hydrophilicity and WHC [25]. According to the above analysis, the most appropriate addition ratio of OVA to LZ was 3:2.

#### 2.1.2. Addition of OEO

As was done above, taking bacteriostasis and water-holding capacity as the main observation indicators, in order to determine the amount of OEO added to the composite gel, this test varied the amount of OEO added to the above composite gel (the ratio of OVA and LZ was 3:2) as 5%, 10%, 15% and 20%, respectively, and the above composite gel samples without OEO were chosen as the blank control. Figure 1C shows the effect of the amount of OEO added on the bacteriostasis of the composite gel. With the increased amount of OEO added, the inhibition of the sample on both bacteria was relatively significant, which was mainly due to the strong antibacterial activity of the carvacrol and thymol present within the OEO [11]. Figure 1D shows the effect of the addition of OEO on the water-holding capacity of the composite gel: with an increased amount of OEO, the water-holding capacity of the composite gel continued to increase, and when 20% OEO was added, the maximum water-holding capacity of the gel reached 67.32%. This might be because the increase in OEO contributed to the formation of flocculation between oil droplets, which limited the flow of water droplets in the composite gel and increased its water retention [26]. Therefore, it is optimal to select 20% OEO for the preparation of antibacterial gel.

### 2.2. Effect of Microwave Treatment on the Hardness of LZ-OEO Antibacterial Gel

**Figure 2:**
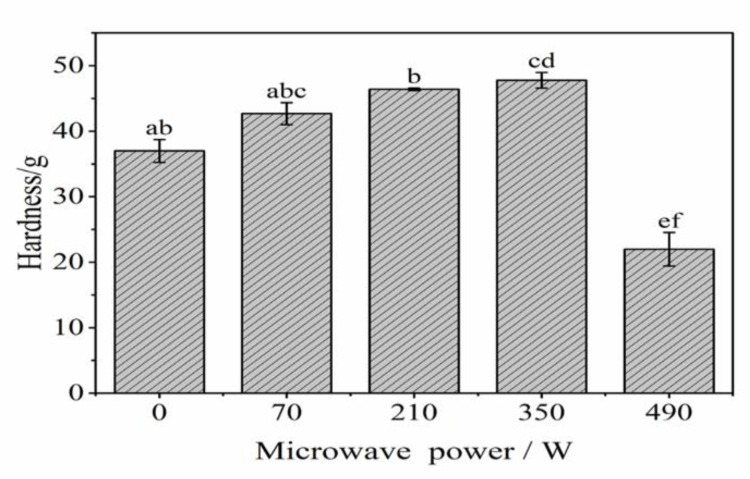
Effect of microwave power on the hardness of lysozyme-oregano essential oil (LZ-OEO) bacteriostatic gels. Different letters of a–f refer to statistically significant differences among the values (*p* < 0.05).

Hardness is one of the important indicators of gel properties. Figure 2 shows that whenever the microwave power was in the range of 0 and 350 W, the hardness of the LZ-OEO gel gradually increased with increased microwave power and reached the maximum hardness at 350 W, which indicates that proper microwave treatment can increase the hardness of gels. This might be because the increase in microwave power can increase the gel temperature, break the covalent bonds between protein molecules, unfold the structure of protein and then help to form a dense and regular network between proteins, therefore enhancing the hardness of samples [27]. However, when the microwave power went too high, the hardness of antibacterial gels decreased significantly (*p* < 0.05). This was probably due to the rapid high temperature induced by excessive microwave power, which promoted the degradation of inulin, shortened the molecular chain and weakened the network structure of gel and thus reduced its hardness [26].

### 2.3. Effect of Microwave Treatment on the Moisture of the LZ-OEO Antibacterial Gel

#### 2.3.1. Water-Holding Capacity of the Gel

**Figure 3:**
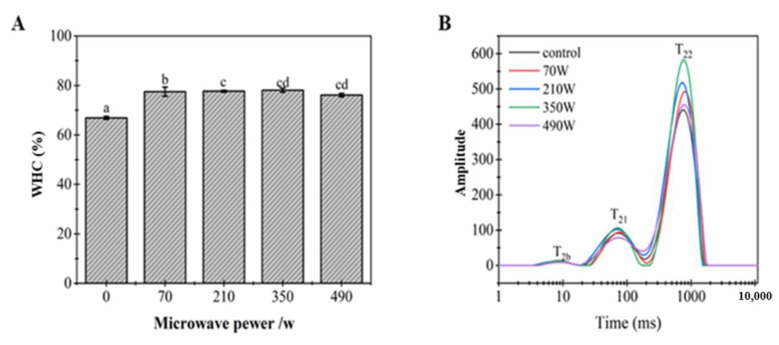
Effect of microwave power on the WHC of lysozyme-oregano essential oil (LZ-OEO) bacteriostatic gels (**A**) and the lateral relaxation curve (**B**). Different letters of “a”, “b”, “c” and “cd” refer to statistically significant differences among the values (*p* < 0.05).

Water-holding capacity is considered to be an important property of gels, which reflects the interaction between protein and water molecules [28]. Figure 3A shows the effect of microwave power on the WHC of the LZ-OEO antibacterial gel. Compared with the 66.90% WHC in non-microwave treated samples, the WHC of LZ-OEO antibacterial gel samples after microwave treatment was significantly increased (*p* < 0.05) and reached the highest value of 78.05% at 350 W. This might be because microwave treatment could induce the structure expansion of polysaccharide chains and proteins and increase the cross-linking between polysaccharides and proteins. Therefore, the water molecules in the cross-linked structure of proteoglycans increased and the content of free water decreased, promoting a more stable LZ-OEO antibacterial gelling system [16].

#### 2.3.2. Distribution of the Gel Moisture

The effect of different microwave power treatments on the mobility of water molecules in LZ-OEO antibacterial gel system was investigated by taking the relaxation time detected by LF-NMR as the main observation index of water distribution in the reaction gel. The LF-NMR spectrum showed that there were three states in the LZ-OEO antibacterial gel: bound water, weak bound water and fixed water, and their transverse relaxation times were T_2b_ (1–10 ms), T_21_ (10–100 ms) and T_22_ (100–1000 ms), respectively. The fixed water was an important part of the gel system, which mainly reflected the quality of water interception in the three-dimensional network of gels [29]. As shown in Figure 3B, with an increase in microwave power, there was no significant change observed in T_2b_ and T_21_ or in their corresponding peak areas (*p* > 0.05), indicating that the change of microwave power had no obvious effect on the distribution of bound water inside the gel. It is seen in Table 1 that with the increase in microwave power, the T_22_ peak emergence time of LZ-OEO antibacterial gel exhibited a delayed trend, and the corresponding peak area (A_22_) exhibited a trend of first increasing and then decreasing. When the microwave power was 350 W, A_22_ reached the maximum, indicating that an appropriate microwave treatment can promote the structure of proteoglycans to expand, reduce the free water content and increase the fixed water content, thereby improving the stability of the LZ-OEO antibacterial gel system [16]. This conclusion was consistent with the conclusion of the water-holding rate in Section 2.3.1.

### 2.4. Effect of Microwave Treatment on the Rheological Properties

**Figure 4:**
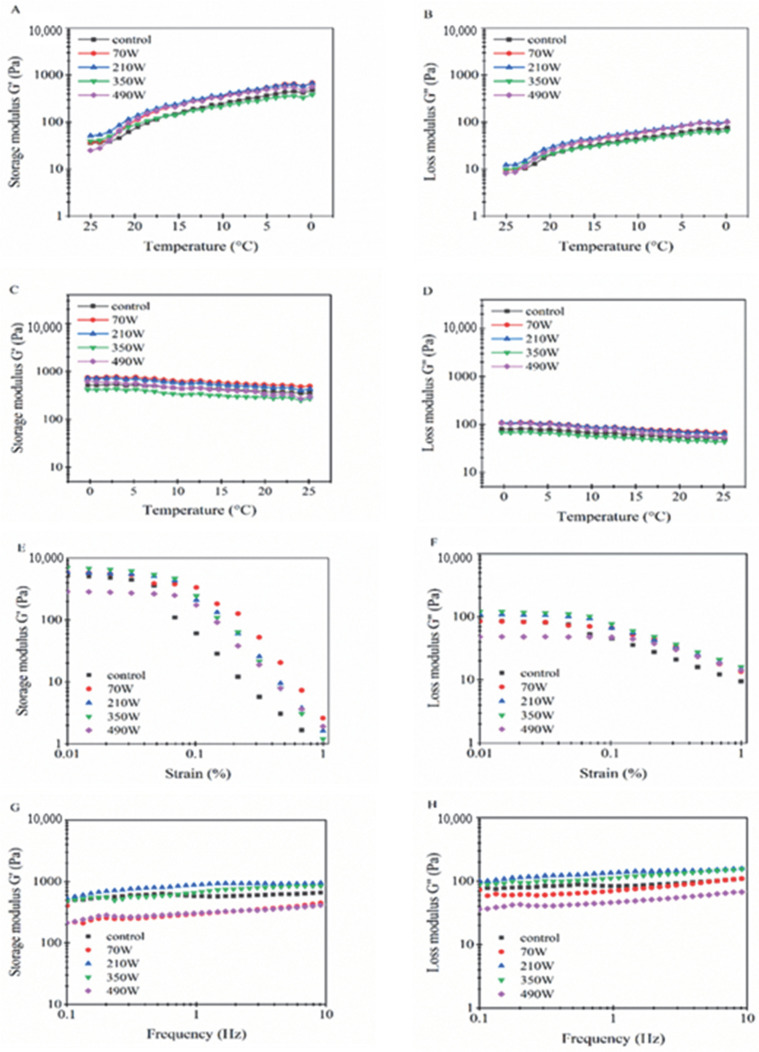
Effect of microwave power on G′ and G′′ of the lysozyme-oregano essential oil (LZ-OEO) bacteriostatic gels during colling scanning (**A**,**B**), heat scanning (**C**,**D**), strain scanning (**E**,**F**) and frequency scanning (**G**,**H**).

Research has shown that the rheological properties of gel are closely related to the structure, stability and functional properties of its system, among which the storage modulus G′ and loss modulus G′′ are two important indicators for characterizing the viscoelasticity of gel [30]. In this study, the effect of microwave power on the viscoelastic properties of the LZ-OEO antibacterial gel was characterized by the dynamic oscillation scanning mode under the cooling and heating cycle. Figure 4A–D shows the change in the rheological curve of G′ and G′′ with time and temperature. The results showed that the volume of G′ was always greater than that of G′′ in the cycle of cooling and heating, which indicates that the sample always maintained the gel consistency. In addition, due to the overall modulus value being low, the LZ-OEO antibacterial gel was easily formed elastic soft gel [16]. In the heating process (Figure 4A,B), G′ and G′′ continued to increase, and it was particularly apparent when the microwave power was 490 W. This indicates that high microwave power was helpful in increasing the viscoelastic properties of the LZ-OEO antibacterial gel. The conclusion might be that the microwave treatment promoted the formation of a gel network structure between particles adsorbed on the oil–water interface and particles not adsorbed in the continuous phase, which then made the gel system more cohesive [31].

The viscoelasticity of LZ-OEO antibacterial gel could be further evaluated by amplitude scanning [30]. The curves of G′ and G′′ changing with strain were shown in Figure 4E,F. Under the condition of low strain amplitude (0.01–0.1%), G′ was greater than G′′, and neither of the changes were obvious. With the gradual increase in the strain amplitude (0.1–1%), both G′ and G′′ began to decrease, and the rate of G′ reduction was significantly higher than that of G′′. This might be because the increase in stress destroyed the particle network structure of LZ-OEO antibacterial gel and improved the viscosity of the gel. The intersection of G′ and G′′ indicates the initial value of structural damage and flow characteristics [32]. With the gradual increase in microwave power, the G′ of LZ-OEO bacteriostatic coagulant showed a trend of first increasing and then decreasing, and G′′ showed a trend of decreasing. G′ was always higher than G′′, indicating that the elasticity of G′ was dominant and larger deformation variables were needed to destroy the gel structure: that is, microwave treatment could strengthen the network structure of the gel system [28]. Among them, the gel system formed under the condition of the microwave set at 350 W was the most stable. This conclusion was consistent with the description on the hardness relationship of bacteriostatic gel mentioned in Section 2.2.

The change curve of G′ and G′′ with frequency is shown in Figure 4G,H. With an increase in frequency, G′ and G′′ both exhibited a slow upward trend. In addition, with an increase in microwave power, the G′ and G′′ of LZ-OEO antibacterial gel increased first and then decreased, especially under the conditions of the microwave set at 350 W where the G′ and G′′ reached the maximum, indicating that the LZ-OEO antibacterial gel with microwave treatment at 350 W had good viscoelasticity.

### 2.5. Effect of Microwave Treatment on the Microstructure of LZ-OEO Antibacterial Gel

#### 2.5.1. Confocal Laser Scanning Microscope (CLSM)

**Figure 5:**
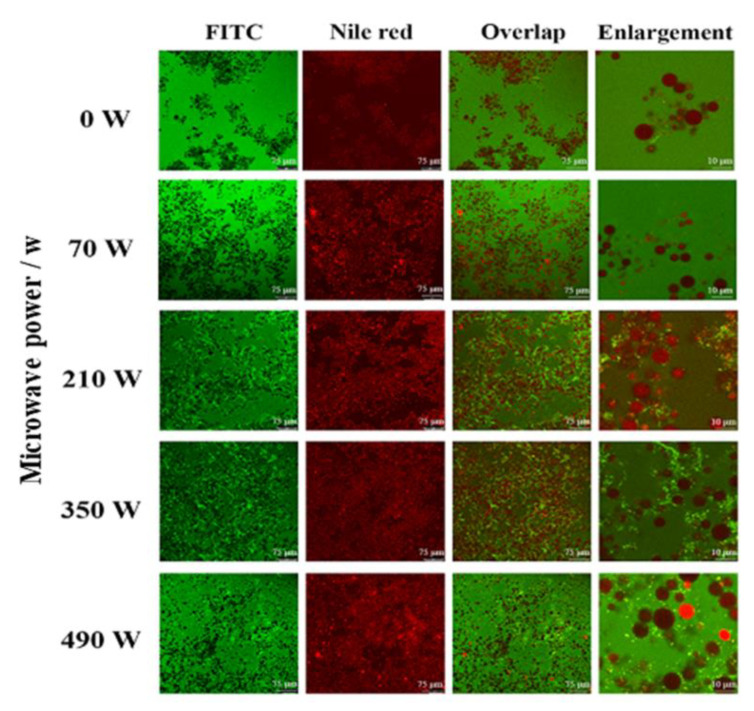
CLSM of the lysozyme-oregano essential oil (LZ-OEO) bacteriostatic gels after microwave treatment. FITC represents isothiocyanine fluorescein; Nile red represents Nile red fluorescein; Overlap represents the overlapping diagram of two fluorescence signals; Enlargement represents a partial enlargement.

The microstructure of LZ-OEO antibacterial gel under different microwave power treatment conditions was observed using a confocal laser microscope, as shown in Figure 5. Green fluorescence was distributed in the dispersed phase (FITC), while red fluorescence was distributed in the oil phase (Nile red), indicating that the two-phase system was an oil-wrapped-in-water emulsion, in which the protein complex was located in the water phase. At the same time, it was discovered that the LZ-OEO antibacterial gel without microwave treatment was more dispersed and had larger particles. The LZ-OEO antibacterial gel after microwave treatment had a uniform system distribution and reduced particle size, which indicated that microwave treatment can improve the distribution characteristics of the LZ-OEO antibacterial gel system and enhance the stability of its network structure [16]. It could be seen from the overlap and enlargement of the two fluorescence signals that with an increase in microwave power, protein molecules were gradually adsorbed on the oil–water interface, and the higher the power was, the more the adsorption would be. In addition, protein molecules and polysaccharides in the aqueous phase formed a gel network structure due to cross-linking, which was distributed around the oil droplets and played a very good role in protecting the volatility of OEO. An explanation might be that the microwave treatment can stretch the protein molecular structure, strengthening the cross-linking between molecules in the aqueous phase [31].

#### 2.5.2. Transmission Electron Microscopy (TEM)

**Figure 6:**
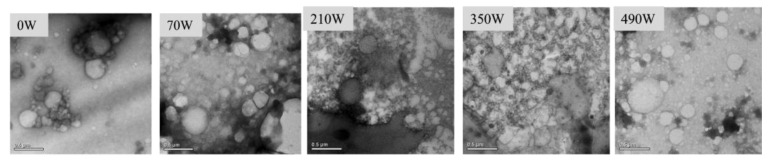
TEM of the lysozyme-oregano essential oil (LZ-OEO) bacteriostatic gels at different microwave power treatments.

The influence of microwave power on the morphology and size distribution of the LZ-OEO antibacterial gel particles is shown in Figure 6. The particles of the LZ-OEO antibacterial gel without microwave treatment were unevenly distributed, accompanied by obvious aggregation. The particle distribution of the LZ-OEO antibacterial gel after microwave treatment was relatively dispersed, especially when the microwave power was 210 W and 350 W. The dense network structure could be seen at the particle interface of the LZ-OEO antibacterial gel, indicating that proper microwave treatment could enhance the binding effect between protein particles and oil droplets [31]. When the microwave power was high, the interface layer of the LZ-OEO antibacterial gel was loose and discontinuous. The reason might be that high microwave power weakens the electrostatic interaction between gel systems and affected its particle cross-linking [26].

### 2.6. Effect of Microwave Treatment on the Antibacterial Property of the LZ-OEO Antibacterial Gel

**Figure 7:**
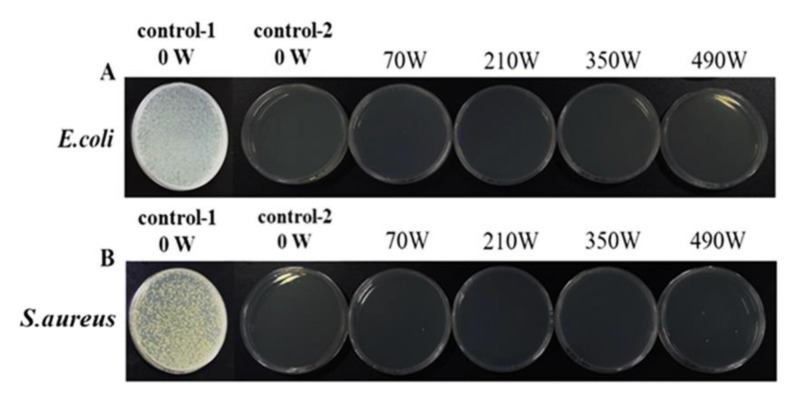
Effect of microwave power on the antibacterial activity of the lysozyme-oregano essential oil (LZ-OEO) bacteriostatic gels: (**A**) *Escherichia coli* (*E. coli*) and (**B**) *Staphylococcus aureus* (*S. aureus*). (Control-1 group was the gel system without bacteriostatic agent and control-2 group was the LZ-OEO bacteriostatic gel system without microwave treatment.)

The effect of microwave treatment on the resistance of LZ-OEO antibacterial gel to *Staphylococcus aureus* and *Escherichia coli* is shown in Figure 7. Compared with the bacteriostasis of the control-1 group, the control-2 group showed a stronger antibacterial effect (*Staphylococcus aureus* and *Escherichia coli*) (*p* < 0.05), indicating that the addition of LZ and OEO can enhance the bacteriostasis of the composite gel. There was no significant difference (*p* > 0.05) between the antibacterial activity of the LZ-OEO antibacterial gel after microwave treatment and that of the gel without microwave treatment, indicating that microwave treatment did not affect the antibacterial activity of the LZ-OEO antibacterial gel system.

## 3. Conclusions

In this study, the proportion of natural bacteriostatic agent added to the lysozyme-oregano essential oil (LZ-OEO) bacteriostatic gel was optimized and the effect of microwave treatment on the performance of LZ-OEO bacteriostatic gel was explored. The results showed that the LZ-OEO antibacterial gel prepared with a ratio of OVA to LZ of 3:2 and the addition of 20% OEO had good water retention and antibacterial properties. Microwave treatment could improve the stability of the LZ-OEO antibacterial gel system. With the increase in microwave power, the hardness of the LZ-OEO antibacterial gel first increased and then decreased. Under the treatment of 350 W microwave power for 1 min, the hardness of the gel reached the maximum the viscoelasticity was the best and the network structure was more compact and orderly. Microwave treatment improved the protective effect of composite gel on OEO, while it did not affect the antibacterial property of LZ-OEO antibacterial gel. This experimental research could provide technical support for the product development and application of natural antibacterial gel, and expand the application ideas of bacteriostatic materials such as lysozyme and oregano essential oil. In the future, it is necessary to focus research on the antibacterial mechanism and influencing factors as well as the specific application scenarios of this material in food and in the chemical industry so as to continuously enhance its industrial value. In addition, since LZ, OVA and inulin are all organic macromolecules, the interaction between the three and whether they have a more effective quality protection and stability effect on other volatile functional essential oils except OEO should be explored.

## 4. Materials and Methods

### 4.1. Materials

*Staphylococcus aureus* (*S. aureus*, ATCC6538) and *Escherichia coli* (*E. coli*, CMCC(B)44102) were purchased from Shanghai Luwei Technology Co., Ltd. (Shanghai, China), lysozyme (LZ) (purity > 98%) was purchased from Sigma-Aldrich, USA (Saint Louis, MO, USA), oregano essential oil (OEO) (purity > 98%) was purchased from Tongze Biotechnology Co., Ltd. (Xi’an, China), Ovalbumin (OVA, biotech grade) was purchased from Macklin Biochemical Co., Ltd. (Shanghai, China), Inulin (analytical grade) was purchased from FANINON Company (Xining, China), Nile red (analytical grade) was purchased from Maclean Biochemical Technology Co., Ltd. (Shanghai, China) and fluorescein isothiocyanate (analytical grade) was purchased from Yeyuan Biotechnology Co. Ltd. (Wuhan, China).

### 4.2. Preparation of Antibacterial Gel

Referring to the preparation method of antibacterial gels of Li et al. with slight modification [26]: 0.6 g OVA, 0.6 g inulin, 0.4 g κ-carrageenan and 18.4 g of ultrapure water were mixed to form liquid A. Meanwhile, 0.2 g κ-carrageenan and 18.4 g ultrapure water were separately mixed with 0, 0.2, 0.3, 0.4 and 0.6 g of LZ, and the corresponding mixed liquids were labelled as liquid B1, B2, B3, B4 and B5, respectively. The above mixed liquids were stirred on a magnetic stirrer for 12 h. Then the liquid A was treated with a microwave oven (70, 210, 350, 490 W) for 1 min. After the liquid A cooled down, equal volumes of liquid A with different powers of microwave treatment were mixed with liquids B1-B5. The mixture of liquid A and liquids B1-B5 without microwave treatment was treated as the control group. Then OEO with the concentration of 0%, 5%, 10%, 15% and 20%, were added to the mixtures, respectively, homogenized at high speed at 10,000 RPM (PT-MR 2100, Kinematica, Switzerland) for 5 min to prepare oil-wrapped-in-water lotion, sealed with cling film, heated in a water bath at 40 ℃ for 90 min, and stored at 4 °C for 12 h for standby.

### 4.3. Determination of Gel Hardness

The hardness of the LZ-OEO antibacterial gel was determined using a TA.XT.plus texture analyzer (Stable Micro Systems, UK), The main principle was to compress the sample twice with the aid of a fixed probe and analyze the hardness parameters through the strength curve of strength with time. The force hardness was determined when the probe reached the compression degree of 30%. The specific test conditions were as follows: the compression degree was 30%, the speed before, during and after the test was 1.0 mm/s, 0.5 mm/s and 1.0 mm/s, respectively, and the trigger force was 0.05 N.

### 4.4. Determination of Water-Holding Capacity (WHC)

An amount of 1 g of a gel sample was wrapped with filter paper, placed in a 10 mL centrifuge tube and centrifuged at 10,000 rpm for 10 min (<10 °C) using a high-speed refrigerated centrifuge (Multifuge, Thermo Fisher Scientific Co., Ltd. China) [33]. The water-holding capacity (*WHC*) was the ratio of mass before and after centrifugation and was calculated with the following equation (all the measurements were repeated three times):

$$WHC=\frac{W_{1}-W}{W_{2}-W}\times 100\%$$

where *W*_1_ represented the sum of the mass of the centrifuge tube and the gel after the water was removed by centrifugation (g), *W*_2_ represented the initial mass of the centrifuge tube and gel (g) and *W* represented the mass of the centrifuge tube (g).

### 4.5. Low Field-Nuclear Magnetic Resonance (LF-NMR)

The LF-NMR relaxation test was determined using the MesoMR23-060V-1 (Niumag Analytical Instrument Co., Ltd. China) nuclear magnetic resonance imaging system. The gel formed after microwave treatment was placed in a cylindrical glass tube with a diameter of 25 mm and T_2_ relaxation time was measured using the CarrePurcelle MeiboomeGill (CPMG) sequence. Probe nmi20-015v-1 was selected and set to echo time, waiting time and scanning times were set as 0.5 ms, 3000 ms and 3, respectively. A total of 4000 echoes were acquired for analysis. Using the simultaneous iterative reconstruction (SIRT) algorithm, the T_2_ relaxation curve was fitted into a multi exponential curve, and the relaxation component and the corresponding area fraction were obtained.

### 4.6. Determination of Rheological Properties

The rheological properties of the LZ-OEO bacteriostatic gel were determined using a HAAKE Rheostress 6000 rotary rheometer (Thermo Fisher Company, Saint Louis, MO, USA). The dynamic rheological behavior of the gel was measured using a 35 mm diameter plate with spacing of 0.5 mm. The measurement sequence was as follows. (1) Temperature scanning: with the help of the dynamic oscillation scanning mode under the cooling heating cycle, the scanning temperature was carried out in the range of 0~25 °C at a heating and cooling rate of 1 °C/min, and the change curves of G′ and G′′ with temperature were recorded. (2) Strain scanning: the strain scanning range was set at 0.01–1% and the scanning frequency was set at 0.1 Hz. The change curves of G′ and G′′ with strain were recorded. (3) Frequency scanning: the strain was set at 0.5%, the frequency scanning was performed within the scanning frequency range of 0.1~10 Hz, and the change curves of G′ and G′′ with frequency were recorded.

### 4.7. Observation of Gel Microstructure

#### 4.7.1. Confocal Laser Scanning Microscope (CLSM)

Nile red (20 μL, 1 mg/mL) and Fluorescein isothiocyanate (FTIC) (20 μL, 1 mg/mL) were thoroughly mixed with 1 mL of the LZ-OEO bacteriostatic gel. Then, the 20 μL mixture was placed on a slide and covered with a coverslip. The excitation wavelengths of the laser (Leica TCS SP8, Leica, Germany) were set at 488 nm and 638 nm under a 20× lens, and the gel morphology was observed.

#### 4.7.2. Transmission Electron Microscope (TEM)

In order to further explore the effect of microwave treatment on the micromorphology of the LZ-OEO bacteriostatic gel, a TecnaiG220 transmission electron microscope (Thermo Fisher Scientific, Amsterdam, The Netherlands) was used to observe TEM images of the LZ-OEO bacteriostatic gel at an accelerating voltage of 80 kV. One drop of LZ-OEO bacteriostatic gel and one drop of phosphotungstic acid (1%, M/V) were mixed and placed on a 200 mesh copper gird for 1 min, and then TEM analysis was conducted [34].

### 4.8. Determination of Bacteriostasis

*S. aureus* (*Staphylococcus aureus* (ATCC6538)) and *E. coli* (*Escherichia coli* (CMCC (B) 44102)) were chosen as the research objects. First, *S. aureus* and *E. coli* were diluted to 5 × 10^8^ CFU/mL and 5 × 10^7^ CFU/mL, respectively. Then 2 mL of *S. aureus* and 2 mL of *E. coli* were mixed with the gels in equal volumes, respectively, and incubated at 37 °C for 48 h in a 200 rpm shaker. After that, the surviving *S. aureus* and *E. coli* in the mixtures were diluted and inoculated into new Petri dishes, respectively, and incubated in the biochemical incubator at 37 °C for 36 h. The growth of *S. aureus* and *E. coli* in the gel were evaluated by colony counting, and the gel not treated by microwave was selected to be the blank control group.

### 4.9. Statistical Analysis

Each experiment was repeated 3 times and the experimental data were expressed by standard deviation (Mean ± SD). Images were drawn using GraphPad Prism 8.03 (GraphPad Software Inc., San Diego, CA, USA) and Origin 2019b (Origin Lab Corporation, Northampton, MA, USA). The GraphPad Prism 8.03 single factor analysis of variance was used for statistical analysis. The *p* value (* *p* < 0.05) represented a significant difference.

## Figures and Tables

**Table 1:** The effect of microwave power on the lateral relaxation time and related peak area of lysozyme-oregano essential oil (LZ-OEO) bacteriostatic gels.

Gel	T_2b_ (ms)	T_21_ (ms)	T_22_ (ms)	A_2b_ (a.u.)	A_21_ (a.u.)	A_22_ (a.u.)
control	7.62 ± 0.83 ^a^	67.80 ± 0.49 ^a^	714.68 ± 39.41 ^a^	107.90 ± 1.07 ^a^	1106.18 ± 9.77 ^a^	5513.08 ± 50.10 ^a^
70 W	8.04 ± 1.05 ^a^	70.52 ± 0.15 ^a^	732.66 ± 49.94 ^a^	126.99 ±1.20 ^ab^	1374.25 ± 29.74 ^b^	6917.94 ± 49.94 ^b^
210 W	8.47 ± 0.27 ^a^	74.02 ± 0.10 ^a^	755.15 ± 45.52 ^a^	136.51 ± 0.10 ^b^	1631.64 ± 19.82 ^c^	7164.39 ± 45.52 ^b^
350 W	8.24 ± 0.25 ^a^	74.02 ± 0.61 ^a^	778.45 ± 34.10 ^a^	137.20 ± 0.10 ^ab^	1669.60 ± 16.93 ^c^	7480.75 ± 34.10 ^b^
490 W	8.18 ± 0.47 ^a^	72.10 ± 0.10 ^a^	755.15 ± 32.41 ^a^	125.28 ± 0.10 ^ab^	1495.41 ± 18.19 ^bc^	7305.04 ± 32.41 ^b^

**Note:** Different letters of ”a”, “b”, “ab”, ”c” and “bc” refer to statistically significant differences among the values (*p* < 0.05).

## Data Availability

Not applicable.
